# Evaluation of three-dimensional reconstructed palatal morphology in skeletal class III subjects with different vertical patterns using cone beam computed tomography

**DOI:** 10.1186/s13005-024-00408-2

**Published:** 2024-01-27

**Authors:** Xiaoyi Huang, Wenbin Huang, Tao Pei, Yijiao Zhao, Yong Wang, Yan Gu, Xueqin Bai

**Affiliations:** 1 Department of Orthodontics, Stomatological Center, Guangdong Provincial High-level Clinical Key Specialty & Guangdong Province Engineering Research Center of Oral Disease Diagnosis and Treatment, Peking University Shenzhen Hospital, Shenzhen, 518036 Guangdong PR China; 2 Department of Materials Science and Engineering, Southern University of Science and Technology, Shenzhen, 518055 Guangdong PR China; 3 Center of Digital Dentistry, Center for Stomatology & National Clinical Research Center for Oral Diseases & National Engineering Research Center of Oral Biomaterials and Digital Medical Devices & Beijing Key Laboratory of Digital Stomatology & NHC Research Center of Engineering and Technology for Computerized Dentistry, Peking University School and Hospital of Stomatology & National, No.22, Zhongguancun South Avenue, Haidian District, Beijing, 100081 PR China; 4 National Center for Stomatology & National Clinical Research Center for Oral Disease & National Engineering Research Center of Oral Biomaterials and Digital Medical Devices, Beijing Key Laboratory of Digital Stomatology, Key Laboratory of Digital Stomatology & Research Center of Engineering and Technology for Computerized Dentistry Ministry of Health & NMPA Key Laboratory for Dental Materials, Department of Orthodontics, Peking University School and Hospital of Stomatology, No. 22 Zhongguancun South Avenue, Haidian District, Beijing, 100081 PR China

**Keywords:** Skeletal class III, Three-dimension, Palatal morphology, Vertical pattern

## Abstract

**Background:**

This study aims to evaluate the difference of three-dimensional (3D) reconstructed palatal morphology between subjects with skeletal Class III and skeletal Class I in different vertical patterns using cone beam computed tomography (CBCT).

**Methods:**

In this study, 89 subjects with skeletal Class III (49 females, 40 males; 25.45 ± 3.81 years) and 85 subjects with skeletal Class I (45 females, 40 males; 23.95 ± 4.45 years) were collected retrospectively and divided into hyperdivergent, normodivergent and hypodivergent groups. Dolphin software was used to reorient the CBCT images of these subjects. After segmenting 3D object of maxilla from the 3D skull by ProPlan software, Geomagic Studio was used to reconstruct 3D palatal morphology and establish an average 3D palatal morphology for each group. The differences of 3D palatal morphology between different groups were compared by deviation patterns on 3D colored map analysis.

**Results:**

3D colored map analysis showed the posterior part of male’s palate was higher and wider than that of female’s palate in skeletal Class III subjects. In skeletal Class III subjects, males with hyperdivergent pattern had a higher and narrower palate compared with hypodivergent subjects, while females with hyperdivergent had a higher but not obviously narrower palate compared with hypodivergent subjects. In the similar vertical patterns, skeletal Class III subjects had a flatter but not narrower palate compared with skeletal Class I subjects, along with a smaller palate volume.

**Conclusions:**

This method allows more intuitive between-group comparisons of the differences of 3D palatal morphology. In skeletal Class III subjects, as the vertical dimension increased, the palate tends to be higher and narrower. Therefore, the influence of vertical patterns on the palatal morphology should be fully considered in the orthodontic and orthognathic treatment of skeletal Class III subjects.

## Background

Skeletal Class III is a common malocclusion caused by both hereditary and environmental factors [1]. Skeletal Class III malocclusion has varying degrees of maxillary retrognathism and/or mandibular prognathism [2]. Retrognathic maxilla with a normal sagittal relationship of mandible was found in 25% of skeletal Class III malocclusion subjects while prognathic mandible with a normal sagittal relationship of maxilla was found in less than 20% of the subjects, and the combination of the two was found in 22% of such a population [3]. Li et al., in their lateral cephalometric sample of 144 Chinese participants with Class III malocclusion, found that 33.3% of their sample had mild mandibular prognathism with a steep mandibular plane while 26.4% had a combination of prognathic mandible and retrognathic maxilla with a flat or normal mandibular plane [4].

In order to formulate a correct orthodontic diagnosis and treatment plan for skeletal Class III patients, previous literature has explored the relationship between palatal morphology and skeletal patterns [5–9]. Franchi and Baccetti investigated the dentoskeletal features of Class III malocclusion using cephalometric analysis and thin-plate spline (TPS) morphometric analysis applied to posteroanterior cephalograms and found that maxillary width was smaller in Class III subjects compared with Class I subjects [6]. Chen et al. also reported dental arch width and maxillary skeletal base width in Class III subjects were significantly smaller than those in Class I subjects by measuring posteroanterior cephalograms [7]. In addition, Chen et al. analyzed dental arch and maxillary skeletal base in Class III malocclusion with different vertical skeletal patterns, concluding that the high-angle group had narrower palates than the low-angle group [8].

However, by the traditional two-dimensional (2D) cephalometry, these analyses of palatal morphology based on angular and linear measurements were insufficient. Recent advances in three-dimensional (3D) technology, such as digital cast and cone beam computed tomography (CBCT), have vastly promoted the exploration of the relationship between 3D palatal morphology and skeletal patterns. Ahn et al. used CBCT to obtain 3D craniofacial skeletal morphology and used digital cast to obtain 3D coordinates of the palate for principal component analysis, and then constructed structural equation modeling (SEM) to analyze the relationship between palate morphology and skeletal patterns [10]. The authors pointed out that as the facial width of skeletal Class III subjects increases, the palate becomes narrower, deeper and longer. Furthermore, Palaoni et al. also collected the 3D coordinates of the palate via digital cast and the measurements of skeletal patterns obtained by lateral cephalograms, using geometric morphometric method (GMM) to analyze the correlation between palatal morphology and skeletal patterns in Class III growing patients. The results revealed that for Class III subjects, increments of mandibular plane angle are related to a narrow and high palate [11]. However, these studies ignored the differences of palatal morphology between males and females, and to some extent, had limitations in describing palatal morphology by specific points on the digital cast, which could not intuitively show the differences in different regions of the palate.

With the development of digital technology, Huang et al. used CBCT to reconstruct 3D palatal morphology and obtained the average palatal morphology within the group to intuitively reflects the differences of 3D palatal morphology between skeletal Class II subjects with retrusive mandible and skeletal Class I subjects in different vertical patterns [12]. However, this method has not been used to analyze the 3D palatal morphology of skeletal Class III patients with different vertical skeletal patterns.

Therefore, the aim of this study was to describe the 3D average palatal morphology of skeletal Class III subjects in different vertical patterns, using CBCT and digital software (e.g. ProPlan (Materialise, Leuven, Belgium), and Geomagic Studio (Durham, NC, US) software), which will provide references for orthodontists and orthognathic surgeon to design clinical plans and evaluate prognosis.

## Methods

### Subjects

In this retrospective study, 89 subjects with skeletal Class III (49 females and 40 males; mean age 25.45 ± 3.81 years) and 85 subjects with skeletal Class I (45 females and 40 males; mean age e 23.95 ± 4.45 years) were recruited from the Department of Oral Maxillofacial Surgery and Department of Orthodontics, Peking University Shenzhen Hospital, and Peking University School and Hospital of Stomatology. This study was approved by Biomedical Ethics Committee of Peking University Shenzhen Hospital and Peking University School and Hospital of Stomatology (Number: PKUSZ-2023-065 and PKUSSIRB-201946086).

### Inclusion and exclusion criteria

The inclusion criteria for skeletal Class I subjects were: (1) Mongolian, (2) aged 18–35 years, (3) 78.8°< SNA < 86.8°, 76.2°< SNB < 84°, 0.7°< ANB < 4.7°, 81.7°< NP-FH < 89.1°(according to Chinese cephalometric norms), (4) no previous orthodontic or orthognathic treatment. Skeletal Class III subjects with ANB < 0.7°and NP-FH > 89.1°were also enrolled in this study. Exclusion criteria for both groups included: (1) missing permanent teeth, (2) retained deciduous teeth, (3) impacted teeth, (4) severe periodontitis, (5) history of palatal surgery, (6) cleft lip and/or palate, (7) craniofacial syndromes, (8) history of mouth breathing and digit sucking.

### Groups

Both skeletal Class III subjects and skeletal Class I subjects were classified into three different sub-groups based on the values of SN-MP, FH-MP, and S-Go/N-Me: hyperdivergent (SN-MP > 37.7°, FH-MP > 32°, S-Go/N-Me < 62%), normodivergent (27.3° < SN-MP < 37.7°, 22°< FH-MP < 32°, 62% < S-Go/N-Me < 68%), and hypodivergent (SN-MP < 27.3°, FH-MP < 22°, S-Go/N-Me > 68%). Table 1 provided the descriptions of the six groups (Class III-hype, Class III-norm, Class III-hypo, Class I-hype, Class I-norm, and Class I-hypo) .

**Table 1:** Description of the subjects

	Skeletal Class III						Skeletal Class I					
	Hyperdivergent		Normodivergent		Hypodivergent		Hyperdivergent		Normodivergent		Hypodivergent	
	Male	Female	Male	Female	Male	Female	Male	Female	Male	Female	Male	Female
n	8	16	15	15	17	18	11	15	14	15	15	15
Age(y)	26.25 ± 2.87	25.38 ± 2.50	25.33 ± 3.02	24.67 ± 3.94	25.47 ± 4.47	25.89 ± 5.10	22.58 ± 3.15	25.87 ± 4.55	22.29 ± 4.23	22.60 ± 3.18	24.80 ± 5.13	25.20 ± 4.97
SNA(°)	78.00 ± 3.86	78.61 ± 2.42	79.45 ± 3.32	79.39 ± 3.60	82.20 ± 3.64	81.49 ± 3.32	80.35 ± 1.11	82.05 ± 1.87	81.51 ± 2.20	81.70 ± 1.88	82.02 ± 2.20	81.82 ± 2.02
SNB(°)	83.69 ± 3.49	82.45 ± 3.57	84.67 ± 3.12	83.49 ± 3.57	88.55 ± 3.85	86.32 ± 2.93	77.46 ± 0.49	78.55 ± 1.57	78.95 ± 1.92	79.13 ± 1.50	79.21 ± 2.17	79.11 ± 2.09
ANB(°)	-5.71 ± 3.95	-3.86 ± 2.15	-5.20 ± 4.37	-4.09 ± 2.43	-6.35 ± 2.42	-4.84 ± 2.47	2.89 ± 1.31	3.49 ± 0.85	2.56 ± 1.18	2.53 ± 1.16	2.81 ± 1.01	2.71 ± 1.12
NP-FH(°)	93.83 ± 2.55	92.04 ± 2.85	95.23 ± 4.07	92.73 ± 2.95	97.19 ± 3.70	95.18 ± 4.02	86.23 ± 2.17	85.99 ± 1.80	86.09 ± 1.28	87.62 ± 1.45	87.59 ± 1.34	87.49 ± 1.34
SN-MP(°)	32.96 ± 1.11	33.89 ± 2.37	26.85 ± 3.21	26.03 ± 3.03	17.89 ± 3.51	18.64 ± 4.76	34.65 ± 2.01	35.39 ± 2.74	28.71 ± 2.06	28.03 ± 2.63	19.04 ± 2.66	18.79 ± 3.72
FH-MP(°)	39.15 ± 0.71	40.04 ± 3.12	33.27 ± 2.26	31.61 ± 2.11	22.46 ± 4.07	23.49 ± 4.24	39.92 ± 2.38	39.82 ± 2.35	31.61 ± 2.27	32.33 ± 2.49	23.03 ± 3.42	23.16 ± 4.06
S-Go/N-Me(%)	60.10 ± 1.62	59.56 ± 1.83	65.85 ± 1.97	65.49 ± 1.51	73.43 ± 3.18	72.09 ± 3.77	60.71 ± 1.84	60.11 ± 2.13	67.01 ± 1.65	66.11 ± 1.42	73.90 ± 3.31	73.55 ± 4.02

*SNA*, Angle formed by sella-nasion-A-point; *SNB*, angle formed by sella-nasion-B-point; *ANB*, angle formed by A-point-nasion-B-point; *NP-FH*, angle between the nasion-pogonion line and frankfort horizontal plane; *SN-MP*, angle between the sella-nasion line and mandibular plane; *FH-MP*, angle between frankfort horizontal plane and mandibular plane; *S-Go/N-Me*, ratio of the distance of sella-gonion to the distance of the nasion-menton

Values are presented as mean ± standard deviation

Sample size calculation was based on palatal height in Huang’s study [12]. PASS software (version 11, NCSS, Kaysvile, Utah) was used to calculate the sample size, and a minimum sample size of 8 subjects were required per group to achieve a significant analysis, with significance level of 0.05 and statistical power of 90%.

### CBCT

The subjects were taken CBCT images before orthodontic or orthognathic treatment using NewTom Scanner (NewTom AG, Marburg, Germany) under the same conditions (axial slice thickness, 0.3 mm; field of view, 15 × 15 cm; scan time, 3.6 s; tube voltage, 110 kV; tube current, 2.81 mA). Dolphin 3D Imaging software (version 11.8, Dolphin Imaging and Management Solutions, Chatsworth, Calif) was used to generate lateral cephalograms from CBCT images.

**Fig. 1:**
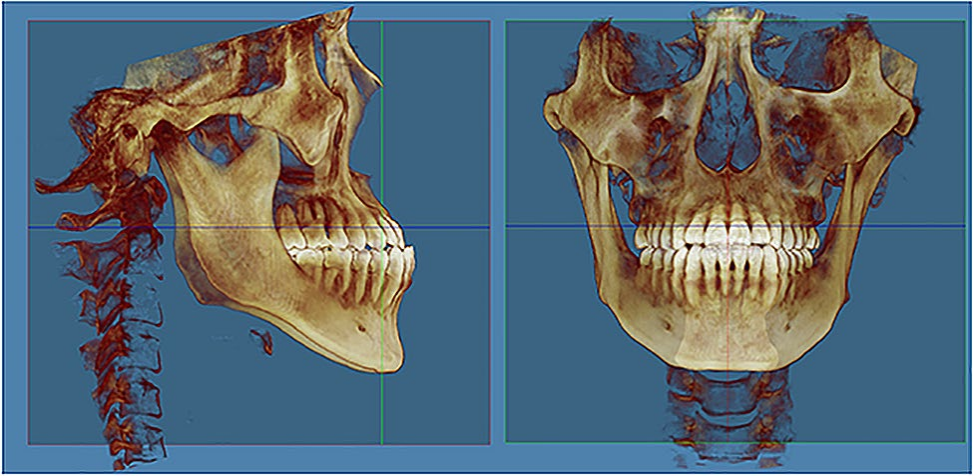
Reorientation for the reconstruction of the three-dimensional (3D) palatal morphology. Horizontal plane, the plane tangent to the most inferior slice of the maxillary alveolar bone; sagittal plane, the plane passing through the ANS-PNS line and perpendicular to the horizontal plane; coronal plane, the plane perpendicular to the above two planes

In order to reconstruct 3D palatal morphology and compare it among different groups, CBCT images were reoriented by the identical 3D reference plane and exported as digital imaging and communications in medicine (DICOM) format by Dolphin software. The plane tangent to the most inferior slice of maxillary alveolar bone was defined as the horizontal plane. The plane passing through the ANS-PNS line and perpendicular to the horizontal plane was defined as the sagittal plane. The plane perpendicular to the above two planes was defined as the coronal plane [13, 14] (Fig. 1).

### 3D palatal morphology reconstruction and measurements

**Fig. 2:**
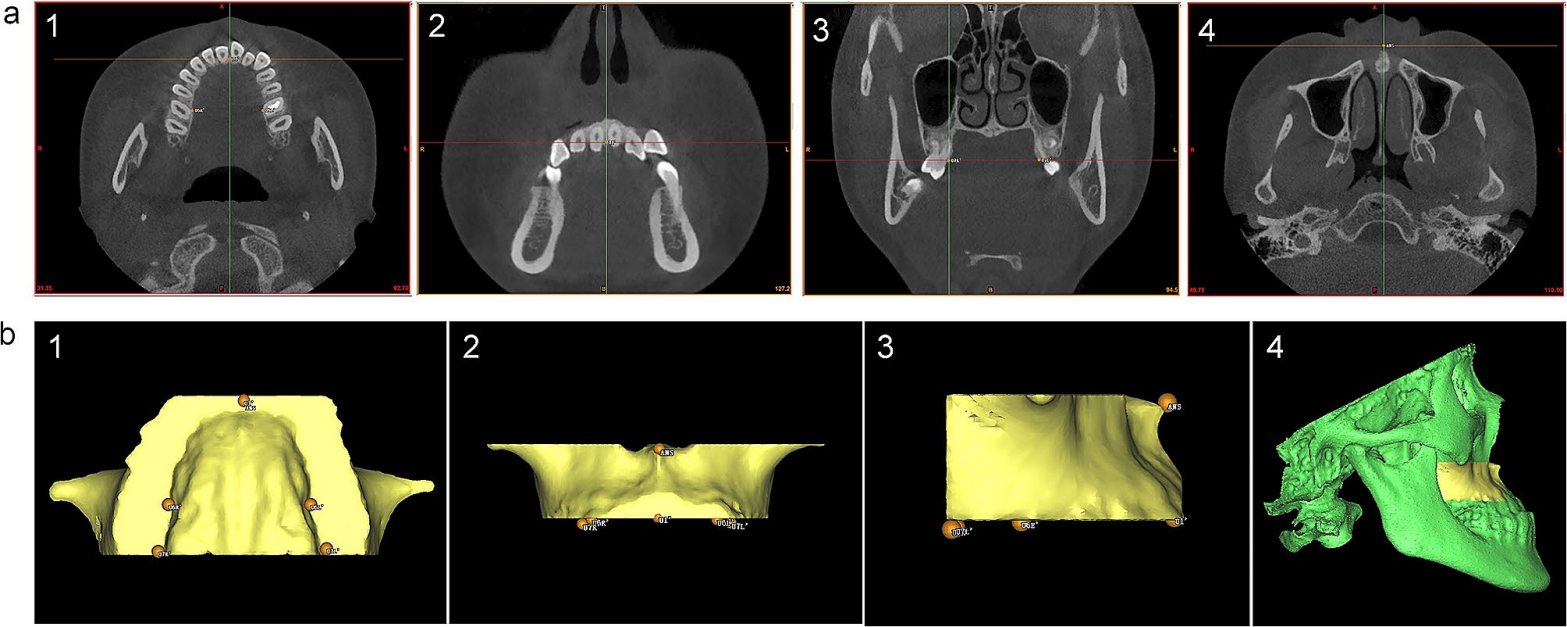
The 3D maxilla obtained from the 3D skull. **a**, the boundaries of the maxilla. 1, The lowermost horizontal plane was through U1’, U6L’, and U6R’; 2, the foremost coronal plane was through U1’; 3, the backmost coronal plane was through U7L’or U7R’; 4, the uppermost horizontal plane was through ANS. **b**, The 3D objects of maxilla. 1, Upwards view; 2, front view; 3, lateral view; 4, the 3D maxilla in the 3D skull

**Fig. 3:**
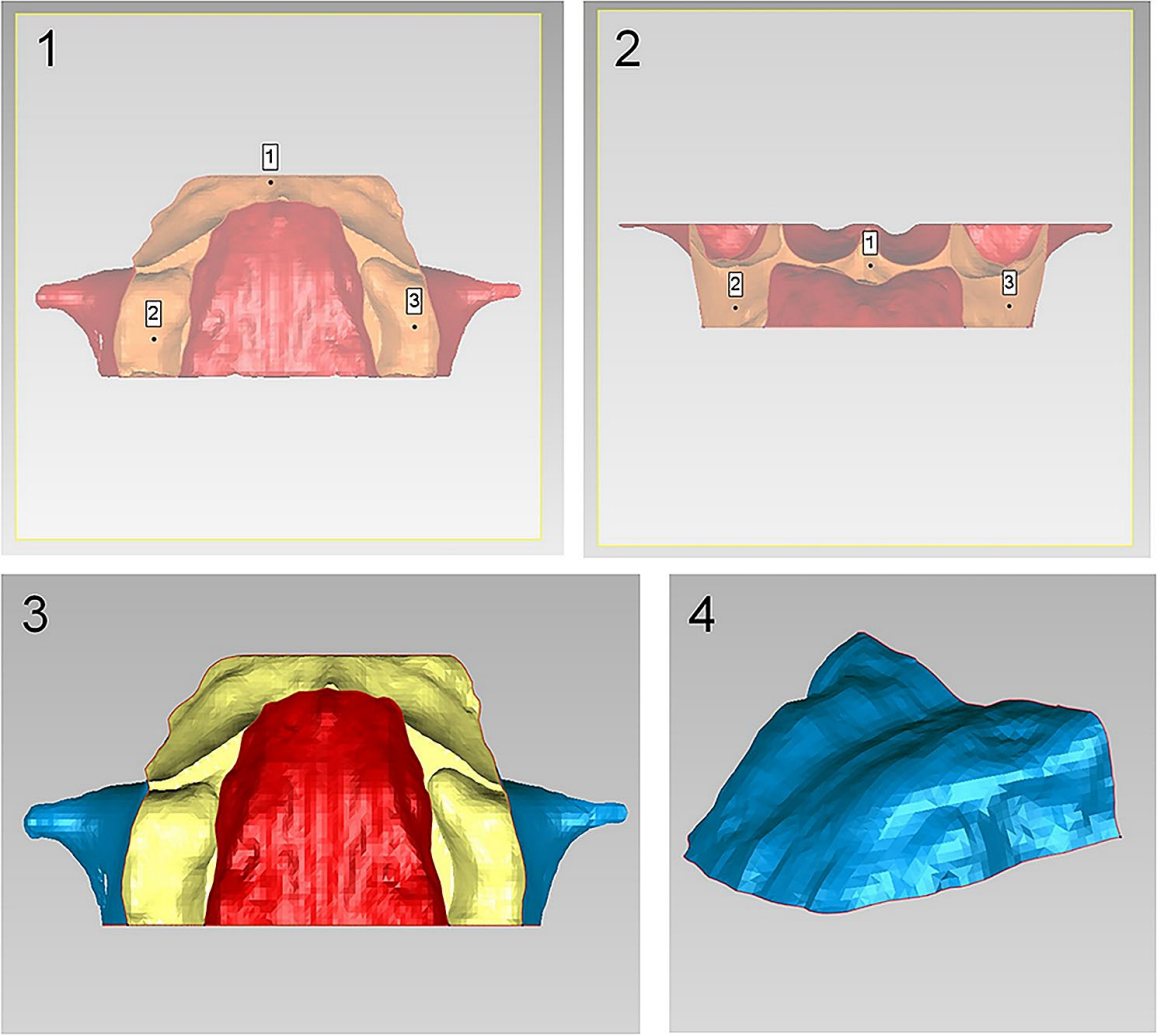
The 3D palatal morphology obtained from the 3D maxilla. 1 and 2, The plane cutting of the lowermost horizontal plane and backmost coronal plane of the maxilla; 3, the palate was selected to create abounded component; 4, the 3D closed figure of the palate was obtained by filling the boundary hole

Firstly, a 3D skull was obtained from reoriented CBCT images by threshold segmentation using ProPlan CMF 1.4 software (Materialise, Leuven, Belgium) [15]. Secondly, to separate the maxilla from the 3D skull, the landmarks of maxillary boundaries were defined as follows: the lowest point on the temporal side of the maxillary anterior alveolar ridge on the median was U1’; the lowest points on the middle of the temporal alveolar ridge of the left and right maxillary first molar were U6L’ and U6R’, respectively; the farthest and lowest points on the temporal alveolar ridge of the left and right maxillary second molar were U7L’ and U7R’, respectively. According to these landmarks, the boundaries of the maxilla were assessed as below: the lowermost horizontal plane was through U1’, U6L’, and U6R’; the foremost coronal plane was through U1’; the backmost coronal plane was through U7L’or U7R’; and the uppermost horizontal plane was through ANS (Fig. 2a). The 3D object of maxilla was segmented and exported as standard tessellation language (STL) format document (Fig. 2b). Finally, Geomagic Studio 11.0 software (Raindrop Geomagic, Inc., NC, USA) was used to transfer reconstructed 3D object of the maxilla into 3D palatal morphology. In order to separate the palate from the maxilla, the lowermost horizontal plane and the backmost coronal plane of the maxilla were chosen through the method of plane cutting. After the separation, the palate was selected to create a bounded component followed by filling the boundary hole to obtain a 3D closed figure of the palate (Fig. 3) [12].

**Fig. 4:**
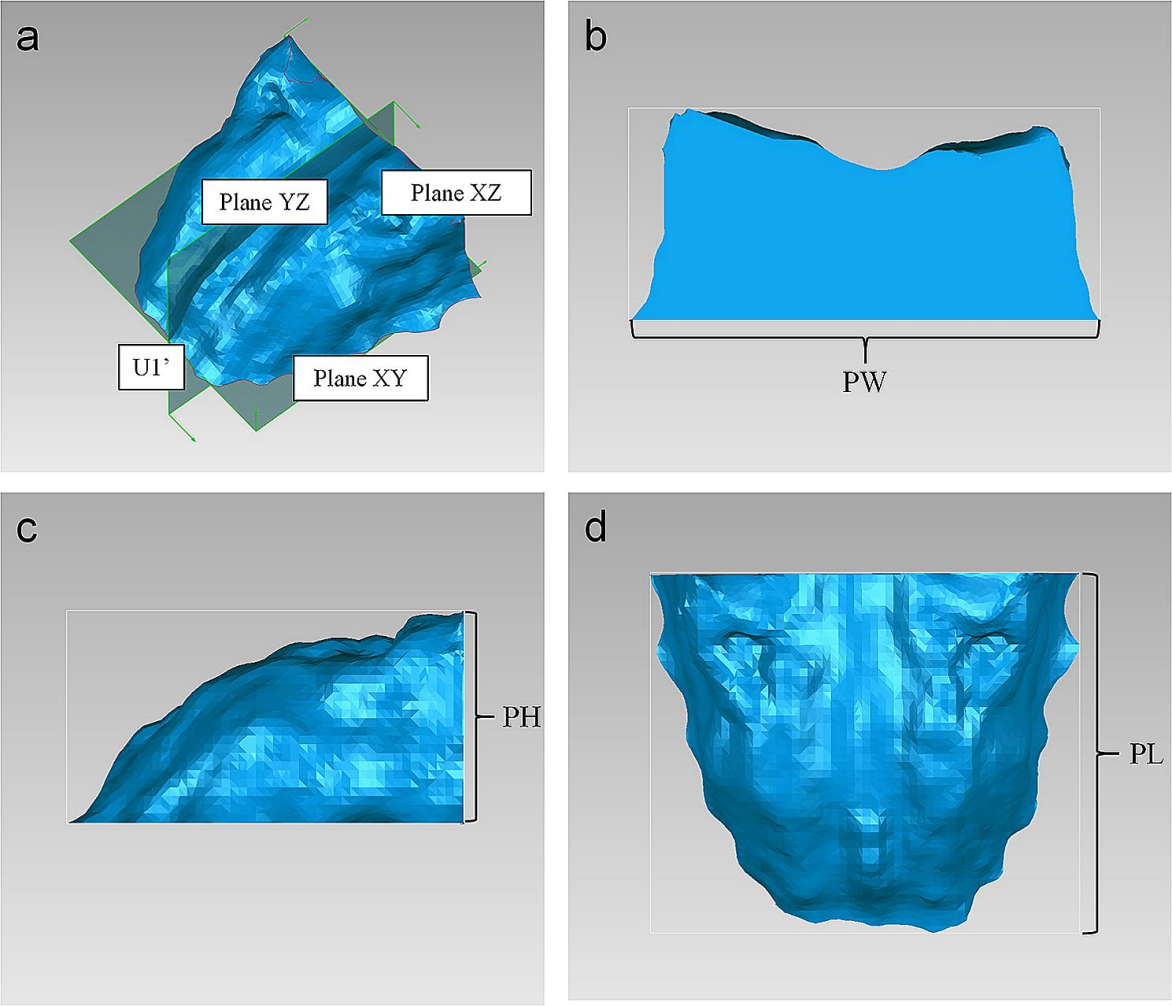
The 3D coordinate system and measurements of the 3D palatal morphology. **a**, The 3D coordinate system of the 3D palatal morphology. U1’, The origin; Plane XY, the horizontal plane; Plane YZ, the sagittal plane; and Plane XZ, the coronal plane. **b**, Palatal width (PW) was measured as the width of the bounding box. **c**, Palatal height (PH) was measured as the height of the bounding box. **d**, Palatal length (PL) was measured as the length of the bounding box

Before obtaining the average 3D palatal morphology of each group, the 3D coordinate system was set as below: the origin was U1’; plane XY was the horizontal plane passing through U1’; plane YZ was the sagittal plane passing through U1’; and plane XZ was the backmost coronal plane perpendicular to the above two planes (Fig. 4a). Then all 3D palatal models were put into this coordinate system, and the method of average calculation was used to establish an average 3D palatal morphology for each group [12].

In addition, Geomagic studio was used to calculate the volume of the 3D closed figure of the palate defined as palatal volume (PV), and the surface area of the 3D closed figure of the palate as palatal area (PA). The width, height, and length of the bounding box were measured as palatal width (PW), palatal height (PH), and palatal length (PL), respectively. (Fig. 4b, c, d) [12, 16].

### Statistical analysis

SPSS software (ver. 23.0, IBM, Armonk, NY, USA) was used to perform the statistical analysis. To evaluate the inter- and intra-observer reliability of the method, 20 CBCT images were randomly selected and 3D palatal shape was re-established by two authors (Xiaoyi, Huang and Wenbin, Huang) at a 2-week interval. Pearson’s correlation was applied to calculate the intraclass correlation coefficient (ICC). Deviation patterns on 3D colored map analysis were performed to evaluate the comparison of 3D palatal morphology among different groups using Geomagic studio. Root mean square estimate values (RMSE) was used to assess the difference values in the comparison of 3D palatal morphology between different vertical pattern groups. Independent 2-sample *t*-test was performed to analyze the differences of PV and PH among different sagittal and vertical pattern groups.

## Results

Table 2 showed that ICC values of PV, PA, PW, PH and PL for both intra- and inter-observer reliability were larger than 0.80, indicating acceptable reproducibility of this method.

**Table 2:** Intraclass correlation coefficients (ICCs) of measurements of 3D palatal morphology

	Intraobserver	Interobserver
PV	0.954	0.969
PA	0.923	0.953
PW	0.824	0.882
PH	0.953	0.975
PL	0.937	0.955

*PV*, palatal volume; *PA*, palatal area; *PW*, palatal width; *PH*, palatal height; and *PL*, palatal length

### Gender difference of 3D palatal morphology in skeletal class III subjects

**Fig. 5:**
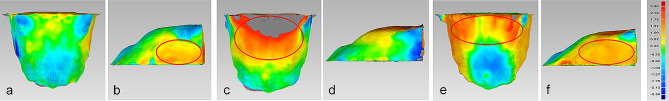
The comparison of 3D palatal morphology in different genders in skeletal Class III subjects. Deviation within 0.25 mm marked in green, ≥2.50 mm marked in red, ≤ -2.50 mm marked in dark blue. Red circle represents markedly positive deviation. Positive deviation means male’s palate was larger than the female’s. a and b, The deviation pattern in Class III-hype group. c and d, the deviation pattern in Class III-norm group. e and f, the deviation pattern in Class III-hypo group

In Class III-norm and Class III-hypo groups, the posterior part of male’s palate was higher than that of female, with a difference of 0.62–2.50 mm, while in Class III-hype group, the posterior part of male’s palate was not significantly higher than that of female (Fig. 5a, c,e). As for the width of the palate, male’s palate was wider than that of female in the posterior part in Class III-hype and Class III-hypo groups, and the differences were both 0.62–1.75 mm, while the posterior part of male’s palate was not significantly wider than that of female in Class III-norm group (Fig. 5b, d, f).

### Comparison of 3D palatal morphology among different vertical patterns in skeletal class III subjects

**Fig. 6:**
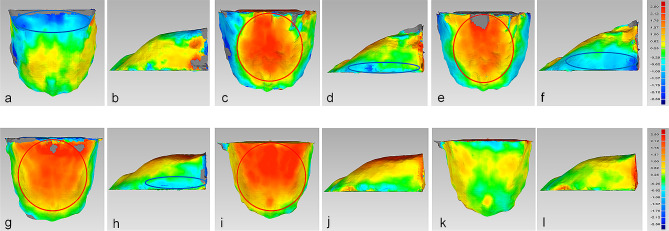
The comparison of 3D palatal morphology in skeletal Class III subjects in various vertical patterns. Deviation within 0.25 mm marked in green, ≥2.50 mm marked in red, ≤ -2.50 mm marked in dark blue. Red circle represents markedly positive deviation, while blue circle represents markedly negative deviation. a-f, The deviation pattern in male. g-l, The deviation pattern in female. a, b, g and h, Positive deviation means palate of Class III-hype subjects was larger than Class III-norm subjects, while negative deviation means palate of Class III-hype subjects was smaller than Class III-norm subjects. c, d, i and j, Positive deviation means palate of Class III-hype subjects was larger than Class III-hypo subjects, while negative deviation means palate of Class III-hype subjects was smaller than Class III-hypo subjects. e, f, k and l, Positive deviation means palate of Class III-norm subjects was larger than Class III-hypo subjects, while negative deviation means palate of Class III-norm subjects was smaller than Class III-hypo subjects

In males, the posterior part of the palate in Class III-hype group was flatter than that in Class III-norm group, and the difference was about 0.62–2.50 mm (Fig. 6a). However, the palate of subjects in Class III-hype and Class III-norm groups were both higher than that in Class III-hypo group, with a difference of 0.62–2.50 mm (Fig. 6c, e). Regarding the width of the palate, it was narrower in Class III-hype and Class III-norm groups than that in Class III-hypo group and the difference were 0.62–1.75 mm and 0.62–2.12 mm, respectively, while the palate in Class III-hype group was not significantly narrower than that in Class III-norm group (Fig. 6b, d, f). In the comparison among these three groups, the maximum RMSE value was 1.14 mm, which was noted in the comparison of Class III-hype and Class III-hypo groups (Table 3).

In females, the palate in Class III-hype group was higher than that in Class III-norm and Class III-hypo groups, and the differences were both about 0.62–2.50 mm (Fig. 6g, i). However, the height of the palate showed no remarkable difference between Class III-norm and Class III-hypo groups (Fig. 6k). In addition, the width of the palate in Class III-hype group showed narrower than that in Class III-norm group, with a difference of 0.62–1.37 mm, while the width of the palate in Class III-hype and Class III-norm groups was also not significantly narrower than that in Class III-hypo group (Fig. 6h, j, l). The maximum RMSE value for the comparison among these three groups was 1.13 mm, which was noted in the comparison of Class III-hype and Class III-hypo groups (Table 3).

**Table 3:** Deviation of comparison of 3D palatal morphology among different vertical patterns in class III groups

		Class III-hype- Class III-norm	Class III-hype- Class III-hypo	Class III-norm- Class III-hypo
Male	Standard deviation(mm)	0.73	1.02	0.91
	RMSE(mm)	0.76	1.14	0.95
Female	Standard deviation(mm)	0.93	0.80	0.82
	RMSE(mm)	0.97	1.13	0.95

*RMSE*, root mean square estimate values

### Comparison of 3D palatal morphology between skeletal class III and skeletal class I subjects

**Fig. 7:**
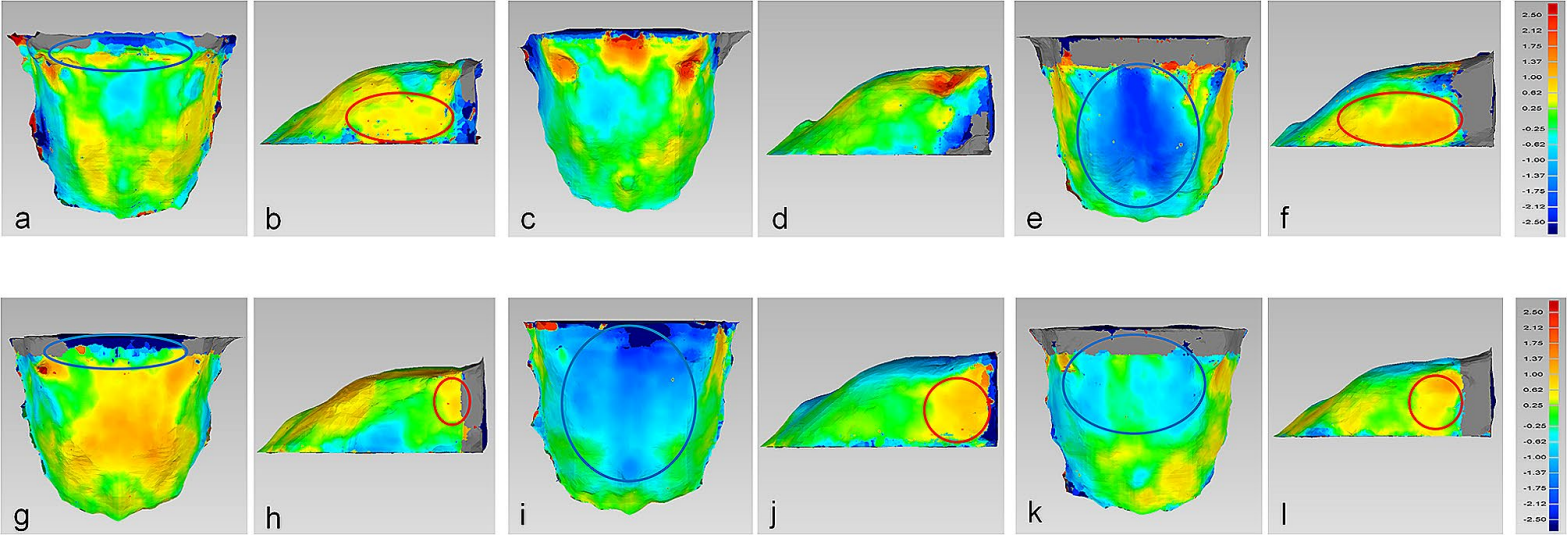
The comparison of 3D palatal morphology in different sagittal patterns. Deviation within 0.25 mm marked in green, ≥2.50 mm marked in red, ≤ -2.50 mm marked in dark blue. Blue circle represents markedly negative deviation. a-f, The deviation pattern in male. g-l, The deviation pattern in female. a, b, g and h, Positive deviation means palate of Class III-hype subjects was larger than Class I-hype subjects, while negative deviation means palate of Class III-hype subjects was smaller than Class I-hype subjects. c, d, i and j, Positive deviation means palate of Class III-norm subjects was larger than Class I-norm subjects, while negative deviation means palate of Class III-norm subjects was smaller than Class I-norm subjects. e, f, k and l, Positive deviation means palate of Class III-hypo subjects was larger than Class I-hypo subjects, while negative deviation means palate of Class III-hypo subjects was smaller than Class I-hypo subjects

In Class III-hype group, the palate was flatter than that in Class I-hype group in the posterior part for both gender, with a difference of 0.62–1.75 mm in males and 0.62–2.50 mm in females (Fig. 7a, g). As for the width, the palate in Class III-hype group was wider than that in Class I-hype group for both gender, and the differences were both 0.62-1.00 mm (Fig. 7b, h). Furthermore, in both males and females, PV of Class III-hype group was smaller than that of Class I-hype group, but the difference was not statistically significant (*p* > 0.05, Table 4).

In Class III-norm group, the female’s palate was flatter than that in Class I-norm group with a difference of 0.62–2.50 mm, while the male’s palate in Class III-norm group showed no remarkable flattening than that in Class I-norm group (Fig. 7c, i). As for the width, the palate in Class III-norm group was wider than that in Class I-norm group in females, with a difference of 0.62–1.37 mm, while the palate in Class III-norm group and Class I-norm group in males showed no significant differences in width (Fig. 7d, j). PV of males in Class III-norm group was significantly smaller than that in Class I-norm group (*p* < 0.05, Table 4).

In Class III-hypo group, the palate was flatter than that in Class I-hypo group for both gender, with a difference of 0.62–2.50 mm in males and 0.25–1.37 mm in females (Fig. 7e, k). Regarding the width, the palate in Class III-hypo group was wider than that in Class I-hypo group for both gender, and the differences were both 0.62–1.37 mm (Fig. 7f, l). In addition, PV in Class III-hypo group was significantly smaller than that in Class I-hypo group (male: *p* < 0.01; female: *p* < 0.01, Table 4). PH of males in Class III-hypo group was significantly smaller than that in Class I-hypo group (*p* < 0.05, Table 4).

**Table 4:** Comparison of two sagittal patterns (class III and class I) with palatal volume and palatal height by independent 2-sample *t* test

		Mean	SD	*p*-value
Male	PV(mm^3^)			
	Class III-hype	9887.31	2406.20	0.267
	Class I-hype	11093.46	2046.11	
	Class III-norm	9865.00	2209.13	0.037*
	Class I-norm	11517.11	1809.19	
	Class III-hypo	7961.54	2205.66	0.000**
	Class I-hypo	10950.78	1426.81	
	PH(mm)			
	Class III-hype	15.42	2.28	0.283
	Class I-hype	16.78	2.78	
	Class III-norm	16.13	2.83	0.787
	Class I-norm	16.38	1.76	
	Class III-hypo	13.95	2.30	0.024*
	Class I-hypo	15.73	1.80	
Female	PV(mm^3^)			
	Class III-hype	9077.30	2131.05	0.268
	Class I-hype	9947.32	2158.18	
	Class III-norm	8450.04	2000.99	0.056
	Class I-norm	9672.40	1275.10	
	Class III-hypo	6980.54	2139.39	0.005**
	Class I-hypo	9046.24	1638.68	
	PH(mm)			
	Class III-hype	15.17	1.99	0.856
	Class I-hype	15.04	2.21	
	Class III-norm	14.08	2.47	0.564
	Class I-norm	14.52	1.55	
	Class III-hypo	12.72	2.52	0.200
	Class I-hypo	13.74	1.83	

*SD*, standard deviation; *PV*, palatal volume; and *PH*, palatal height

* *p* < 0.05, ** *p* < 0.01

## Discussion

With the development of 3D imaging technology, many studies have evaluated the morphological differences of palate from a 3D perspective. Leonardi et al. and Lo Giudice et al. used digital software (Geomagic Qualify software and Geomagic Control™ X) to compare the asymmetry of palatal morphology by obtaining mirrored palate model and superimposing it with the original palate model [17, 18]. Huang et al. used CBCT and digital software to reconstruct 3D palatal morphology, and obtained the average palatal morphology within the group to analyze the differences of 3D palatal morphology in skeletal Class II subjects with retrusive mandible and different vertical skeletal patterns [12]. This method allowed more intuitive between-group comparisons of the differences of 3D palatal morphology. However, this method has not been used to analyze the 3D palatal morphology of skeletal Class III patients with different vertical skeletal patterns. Intuitive analysis of 3D palatal morphology of skeletal Class III patients with different vertical skeletal patterns can help orthodontists and orthognathic surgeon to design clinical plans and evaluate prognosis, which is conducive to obtain more stable treatment results.

In the literature, Ahn et al. obtained the palatal coordinates of the digital model and used SEM to analyze the correlation between palatal morphology and skeletal patterns [10], while Paoloni et al. used GMM to analyze the correlation between palatal morphology and skeletal patterns in Class III growing patients [11]. In addition to acquiring imaging data of the subjects, these methods also need to obtain digital casts through scanning. Besides, previous methods could not obtain the average palatal morphology within the group for direct comparison, and the model reconstructed by specific points on the palate still loses part of the 3D morphological information of the palate. However, in this study, digital software was used to reconstruct the 3D palatal morphology on the basis of CBCT and calculate the average morphology within the group, which retained the 3D morphological information of the palate to the greatest extent. On top of that, the 3D deviation colored map was used to visually display the differences in the palatal morphology between groups. In addition, this study found gender difference in 3D palatal morphology, and analyzed the differences in 3D palatal morphology between skeletal Class III patients with different vertical skeletal patterns in males and females separately.

### Comparison of 3D palatal morphology with different vertical patterns in skeletal class III subjects

In skeletal Class III subjects, both males and females, hyperdivergent subjects had a higher and narrower palate than hypodivergent subjects, which was consistent with the results of previous studies [8, 11, 19, 20]. Palaoni et al. used GMM and found that the vertical patterns had the greatest influence on the palatal morphology in skeletal Class III subjects, and as the mandibular plane angle increased, the palate became higher and narrower [11]. By comparing the width of maxillary base and intermolar on the posteroanterior cephalograms of 50 skeletal Class III subjects aged 10–14 years old with different vertical patterns, Chen et al. concluded that the width of maxillary base and intermolar in hyperdivergent subjects were smaller than those in hypodivergent subjects [8]. In addition, Ning et al. used CBCT to measure the maxillary width of skeletal Class III adults with different vertical patterns, and the results showed that regardless of gender, the maxillary width and alveolar width of hyperdivergent subjects were smaller than those of hypodivergent subjects [20].

This may be due to the differences in the function and morphology of masticatory muscles in subjects with different vertical patterns [21, 22]. Al-Farra et al. evaluated the metabolic differences of the masseter muscle in subjects with different vertical patterns by magnetic resonance spectroscopy (MRS) and found that the inorganic phosphate/phosphocreatine (Pi/PCr) ratio decreased with the increase of the mandibular plane angle, which means that the metabolism of masseter muscle was more active in hypodivergent subjects, and further limited the vertical growth of the mandible [21]. Besides, Biondi et al. used MR to measure the masseter muscle volume, ultrasound (US) to measure the thickness of the masseter muscle, and maxillary cast to measure the maxillary intermolar width [22]. The results showed that with the increase of the mandibular plane angle, the volume and thickness of the masseter muscle decreased, and the maxillary intermolar width also decreased, and there was a significant positive correlation between the maxillary intermolar width and the masseter muscle volume. Thus, the reduced volume and metabolism of the masseter muscle in hyperdivergent subjects is accompanied by a decrease in palatal width, which could partially explain that hyperdivergent subjects had a higher and narrower palate than hypodivergent subjects. In conclusion, the craniomaxillofacial system is a functional complex, and all parts interact with each other. Therefore, the influence of vertical patterns on the palatal morphology should be fully considered in the orthodontic and orthognathic treatment of skeletal Class III subjects. When changing the palatal width, muscle training can be used to improve the treatment stability.

### Comparison of 3D palatal morphology between skeletal class III and skeletal class I subjects

The present study found no significant narrowing of the palatal width in skeletal Class III subjects compared with skeletal Class I subjects in similar vertical patterns, which is inconsistent with the results of previous studies. Franchi and Baccetti and Chen et al. used posteroanterior cephalograms to measure the width of the maxillary base and intermolar, and both found that the width of the maxillary base and intermolar in skeletal Class III subjects were narrower than those in skeletal Class I subjects [6, 7]. With the help of CBCT, Ning et al. measured the maxillary width and maxillary alveolar bone of skeletal Class III subjects and skeletal class I subjects in different gender groups. They found that the maxillary width and maxillary alveolar bone of skeletal Class III subjects were narrower than those of skeletal Class I subjects, regardless of gender [20].

The inconsistent results among previous studies and the current study may be due to the different measurement methods and included samples. First of all, previous studies represented the palatal width by measuring the distance between two points, but the present study reconstructed the 3D palatal morphology by digital methods, which could show the difference in the overall width of the palate between different groups, and yielded more intuitive and reliable results. Secondly, previous studies on the influence of sagittal patterns on palatal morphology did not classify subjects according to their vertical patterns, so the influence of vertical patterns on palatal morphology cannot be excluded. However, Paoloni’s study showed that the vertical patterns had the greatest influence on the height and width of the palate [11]. Therefore, ignoring the effect of vertical patterns on palatal morphology may cause errors in the results. In this study, however, the comparison of palatal morphology between skeletal Class III subjects and skeletal Class I subjects was under similar vertical patterns, which might be more credible.

There are few studies on the palatal height of skeletal Class III subjects in the previous literature. The present study found that in similar vertical patterns, the posterior part of the palate of skeletal Class III subjects was flatter than that of skeletal Class I subjects, along with a smaller palatal volume. Since the palate is a part of the upper airway, appropriate digital technology can be used in the future to further explore the relationship between palatal morphology and upper airway morphology in skeletal Class III subjects [23].

## Conclusion

1. The posterior part of male’s palate was higher and wider than that of female in skeletal Class III subjects.
2. In skeletal Class III groups, hyperdivergent subjects had a higher and narrower palate compared with hypodivergent subjects in males, while in females, the palate of hyperdivergent subjects was higher but not obviously narrower than that of hypodivergent subjects.
3. In the similar vertical patterns, the palate of skeletal Class III subjects was flatter but not narrower than that of skeletal Class I subjects, along with a smaller volume.

## Data Availability

The datasets used and/or analysed during the current study are available from the corresponding author on reasonable request.

## Abbreviations

ANB: Angle formed by A-point–nasion–B-point
CBCT: Cone-beam computed tomography
DICOM: Digital imaging and communications in medicine
FH-MP: Angle between frankfort horizontal plane and mandibular plane
GMM: Geometric morphometric method
ICC: Intraclass correlation coefficient
MRS: Magnetic resonance spectroscopy
NP-FH: Angle between the nasion-pogonion line and frankfort horizontal plane
PA: Palatal area
PH: Palatal height
Pi/PCr: Phosphate/phosphocreatine
PL: Palatal length
PV: Palatal volume
PW: Palatal width
RMSE: Root mean square estimate values
SD: Standard deviation
SEM: Structural equation modeling
S-Go/N-Me: Ratio of the distance of sella-gonion to the distance of the nasion-menton
SNA: Angle formed by sella-nasion-A-point
SNB: Angle formed by sella–nasion–B-point
SN-MP: Angle between the sella-nasion line and mandibular plane
SPSS: Statistical package for social science
STL: Standard tessellation language
TPS: Thin-plate spline
US: Ultrasound
2D: Two-dimensional
3D: Three-dimensional
